# Patient safety in eye care: a multi-method analysis of reported incidents involving implementation of care and clinical assessment in England and Wales

**DOI:** 10.1038/s41433-025-03669-6

**Published:** 2025-02-13

**Authors:** Jennifer H. Acton, Joy McFadzean, Chun Yun Lau, Jih Wenn Foo, Andrew Carson-Stevens

**Affiliations:** 1 School of Optometry and Vision Sciences, Cardiff University, Cardiff, UK; 2 PRIME Centre Wales, Division of Population Medicine, School of Medicine, Cardiff University, Cardiff, UK

**Keywords:** Public health, Health services, Outcomes research, Epidemiology

## Abstract

**Background/objectives:**

Patient safety is a global health priority, yet there is limited research into how ophthalmology is responding to this. There is evidence that a review of patient harm related to eye care and the associated patient safety incidents is needed. We aimed to characterise patient safety incidents involving eye care by: identifying the most frequently reported incidents involving clinical care; and characterising the nature of incidents leading to severe vision loss.

**Methods:**

The data comprised patient safety incidents reported between 2018 and 2022 to the National Reporting and Learning System and the NHS England Learn from Patient Safety Events system. Reports were searched for eye-related terms (ICD-11) and those reports relating to implementation of care and clinical assessment were included. A descriptive analysis was undertaken to characterise the most frequent incident types and their contributory factors, followed by a thematic analysis of incidents relating to severe vision loss.

**Results:**

Of the 836 reports identified, insufficient care (*n* = 416) and delayed diagnosis (*n* = 234) featured most. Patient harm occurred related to vision loss (*n* = 449), delays in treatment (*n* = 182), and disease progression (*n* = 121). Among 220 reports that resulted in severe vision loss, patients with Glaucoma and Age-related Macular Degeneration were impacted by delays in monitoring and management, loss to follow-up, disease progression due to insufficient care and system failures.

**Conclusions:**

In this characterisation of eye-related incident reports in a national population, potential areas of interest toward safer eye care include addressing delays in patients receiving care and insufficient care such as inconsistent monitoring in glaucoma.

## Introduction

The improvement of patient safety is a global health priority, yet there is a paucity of evidence from research in eye care settings. Given the recent developments in eye care pathways in the UK, such as the transition of services from hospital to primary care [1–4], in addition to increasing demand on services, and the declining performance of the National Health Service (NHS [5]; it is essential that a detailed understanding of patient safety incidents in eye care is gained.

There are growing patient safety concerns in healthcare with around one in 20 patients exposed to preventable harm [6] and in primary care, greater levels of harm were associated with diagnostic and medication incidents [6–8]. Yet the prevalence and types of harm in eye care are not well described. Eye-related patient safety remains underrepresented in health-related policy, yet patient safety reporting was deemed a high-priority area for quality improvement related to eye disorders [9]. A limited body of literature on eye-related patient safety includes incidents in primary eye care [10], ophthalmic surgery [11–15], and emergency eye care [16]. The types of incidents reported in eye care resulted from delayed eye care [11, 15–17] and medication errors [11, 13]. For quality of care to improve, a detailed knowledge of the frequency of occurrence and nature of harm in eye care is needed to allow the development of strategies for prevention.

The Learn from Patient Safety Events (LFPSE; previously England and Wales National Reporting and Learning System, NRLS) is the largest database of patient safety incidents in the world and receives more than 2.5 million reports each year from national NHS healthcare providers. The LFPSE or NRLS database has been investigated widely in primary care in general practice [18, 19]; for medication incidents [20, 21]; in hospitals [22]; orthopaedic surgery [23]; dentistry [24]; and mental health [25]. However, the nature and severity of eye-related safety incidents reported through the NRLS and LFPSE are unknown.

The aim of this study was to understand the nature of reported patient safety incidents in eye care. The objectives were firstly to identify the most frequently reported incidents involving the implementation of care and clinical assessment. The second objective was to characterise the nature of those incidents leading to severe vision loss and identify potential areas as to where and how safer care may be delivered within eye care within the reports.

## Methods

### Study design

An exploratory multi-methods analysis of incident reports about eye care was undertaken. The definition of a patient safety incident is “any unintended or unexpected incidents that could have, or did, lead to harm for one or more patients receiving health care” [26]. The use of incident reports as a data source allows an understanding to be gained of the events that occurred and the reporter’s perception as to why. Data coding and exploratory data analysis can be used to identify the most frequently reported incidents [27], in addition to a thematic analysis [28, 29] to determine themes about why incidents occur.

### Study population

The NRLS and LFPSE database contained fully anonymised reports from healthcare professionals, who have a responsibility to report incidents to healthcare organisations. Data held in each report included location, patient demographics, reported severity of harm, and a ‘free-text’ description of the incident and possible contributory factors.

The study population comprised all patient safety incident reports relating to eye care from incidents reported to the NRLS and LFPSE between January 2018 and May 2022. Incidents reported before September 2021 were from England and Wales, and those reported thereafter were from England only. Eye-related reports were identified at the data source through relevant search terms based on the World Health Organization International Classification of Disease codes (ICD-11), encompassing eye and visual system relevant pathology and trauma (see Table S1), and were then shared with the researchers. Codes from ICD-11 were used to encompass a broad range of relevant terminology. The search terms were applied to the free-text description of the incident.

The search results were coded using an empirically developed multi-axial framework, the ‘PatIent Safety (PISA) classification system’, which has been used to analyse over 75,000 incident reports across a range of health and social care contexts [27]. We adopted an internationally established multi-methods approach to generate learning from patient safety incidents [27, 30]. Those incidents categorised under implementation of care and clinical assessment formed the sample for analysis. Incidents relating to implementation of care and clinical assessment (pre-designated categories within the data supplied) were selected based on their encompassing nature as broadly representative of general issues which may occur in the patient journey in eye care. Four categories were chronologically coded according to: incident type, contributory factors, incident outcome and incident severity as reported [27]. Additionally, the incident severity was graded by the researchers according to a classification system aligned with the WHO classification ([31]; Table 1) and previously validated [27, 30].

**Table 1:** Severity of harm described in the incident reports (*n* = 836).

Severity of harm	Definition	Example	Reports n (%)
No harm	Patient outcome is not symptomatic, and no treatment is required.	Humphrey visual field test was not done although found to cause no harm to the patient.	142 (17)
Low harm	Patient outcome is symptomatic, symptoms are mild, loss of function or harm is minimal and intermediate but short term, and no or minimal intervention is required.	The appointment was delayed in a patient undergoing treatment for diabetic retinopathy. Slight progression of retinal pathologic signs were noted, but vision was unaffected.	202 (24)
Moderate harm	Patient outcome is symptomatic requiring intervention, an increased length of stay, or causing permanent or long-term harm or loss of function.	Appointment was delayed. Permanent loss of peripheral visual field, not affecting patient’s central vision.	227 (27)
Severe harm	Patient outcome is symptomatic, requiring life-saving intervention or major surgical/medical intervention, shortening life expectancy, or causing major permanent or long-term harm or loss of function.	Appointment was delayed. Vision dropped from 6/24 to count fingers (CF), had a hypotonous eye and required emergency surgery as a result.	262 (31)
Unclear	It is unclear from the free-text description what level of harm has occurred	Patient underwent treatment for corneal ulcer, and later died from a severe acute kidney infection	3 (<1)

### Analysis

Reports were included if they met the definition of a patient safety incident [26], and contained sufficient information to determine what happened and to determine the causes of the incident. Reports were excluded if they were not related to eye care.

### The analysis of the reports involved 3 stages: [27]

Firstly, familiarisation and data coding consisted of reviewing the free text within each report and applying codes systematically to describe incident type, potential contributory factors, level and type of harm. Codes were applied using a previously derived codebook [30] with the addition of new codes as needed, with consensus in the research team upon implementation. Regular meetings were held to review codes, with arbitration with trained researchers as needed to ensure uniformity of coding.

Secondly, generation of data summaries involved the use of descriptive statistical analysis. Pivot tables in Excel (Microsoft Corp., Redmond, WA, USA) were used to cross-tabulate level of harm with incident type, and contributory factor and incident type.

Thirdly, interpretation of themes and learning, in which patient safety themes were identified and explained by gaining an understanding of the events leading up to it and contributory factors, and specific contexts.

To address the second objective, incident reports were selected for thematic analysis if they included sufficient information to carry out a descriptive analysis; if the outcome for the patient resulted in vision loss; and if they were categorised under the most severe level of harm. The selection was made to gain an understanding of the perceived circumstances which may lead to an outcome that is often perceived as the worst for patients [32]. Free-text data were analysed using NVivo (version 1.7.1; QSR International) to identify and prioritise the safety problems through the identification of recurring themes and subthemes. Patterns in the coding were identified through discussion between the researchers (CYL, JF, JHA) and an iterative approach was adopted to develop the themes with input from the wider team (JM).

The researchers (CYL, JF, JHA) had undergone training in incident analysis, classification, and root cause analysis, including simulation with practice cases, as described previously [27]. One researcher conducted all coding, and a second researcher reviewed a 20% random sample of codes. Inter-rater reliability was calculated using Cohen’s kappa coefficient, with a kappa value of greater than or equal to 0.7 used to indicate moderate-high agreement. Differences in coding were resolved through discussion and arbitration with a senior investigator.

The reporting adheres to the Strengthening the Reporting of Observational Studies in Epidemiology (STROBE) guidelines (Table S2) and the Standards for Reporting Qualitative Research (SRQR; Table S3).

## Results

**Fig. 1:**
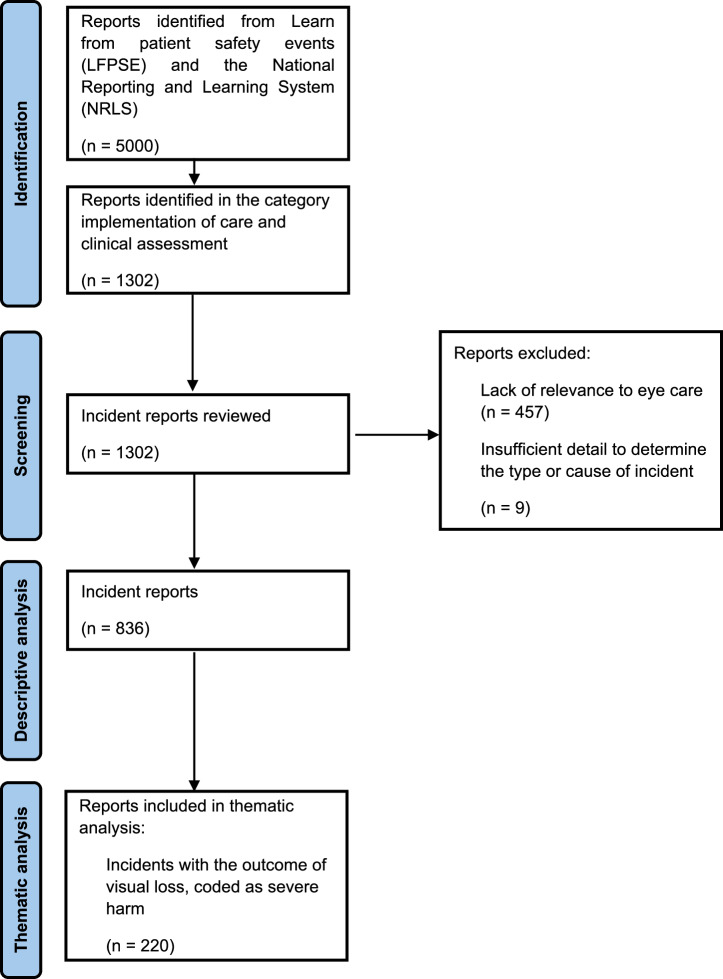
Summary of sample formation.

NHS England provided 5000 reports for processing (Table S4) and following filtering by incident category for implementation of care and clinical assessment, a sample of 1302 reports was identified for analysis. After initial review, 836 were included in the quantitative analysis. Incidents were excluded on the basis of lack of relevance to eye care (*n* = 457) or lack of sufficient detail (*n* = 9; Fig. 1).

Table 1 shows the definitions of harm and the number of reports that describe the level of harm.

A Cohen’s kappa coefficient of 0.85 for inclusion or exclusion of incident reports between two coders, indicated high inter-rater reliability.

### Most frequently reported incident types and outcomes

Insufficient treatment/care/monitoring given (*n* = 416, 50%), delayed diagnosis (*n* = 234, 28%), wrong diagnosis (*n* = 46, 6%), insufficient assessment (*n* = 46, 6%), and implementation issues conducting the correctly chosen process or procedure were most frequently observed among the reports (*n* = 43, 5%; Table 2). The most frequent outcomes reported for patients included vision loss (*n* = 449, 54%), delays in management assessment or treatment (*n* = 182, 22%), and general deterioration/ progression of condition (*n* = 121, 14%). These three outcomes also represented the outcomes with the greatest level of harm (severe harm) reported. Of all the incidents, 41% resulted in either no harm or low harm (*n* = 344), and 59% were assigned a code from low to severe harm (*n* = 492). Incidents with the outcome of vision loss, coded as moderate or severe harm (*n* = 372) were mainly due to insufficient treatment/care/monitoring given (*n* = 194, 52%) or delayed diagnoses (*n* = 122, 33%).

**Table 2:** Incident type by outcome.

### Relationship between incident type and contributory factors

The most frequent contributory factors reported involved poor continuity of care, seen frequently with appointments being cancelled by the hospital without patient’s knowing (*n* = 365, 44%); inadequate skillsets or knowledge related to incidents, such as patients being referred to the wrong clinics (*n* = 222, 27%); and long waits for service, such as delayed appointments (*n* = 51, 6%). The relationships incident type and contributory factors are shown in Table 3.

**Table 3:** Distribution of all possible combinations of incidents and contributory factors.

Reported issues with continuity of care featured the most when insufficient treatment/care/monitoring occurred (*n* = 274, 66%). Whilst an inadequate skill set or knowledge, and continuity of care, contributed the most to delayed diagnoses (*n* = 95, 41% and *n* = 64, 27%, respectively) within reports.

Interestingly, among incidents reported involving insufficient treatment/care/monitoring, only 26 (6%) incidents were perceived to be related to the COVID-19 pandemic.

### Themes from incidents resulting in the most serious harm in which vision loss occurred

Of the 836 incident reports, 220 incident reports were selected for thematic analysis on the basis of those with the greatest severity of harm in which vision loss occurred.

Three key themes were identified (Table 4, additional themes in Table S5).

**Table 4:** List of themes from incidents resulting in the most serious harm in which vision loss occurred and additional example quotes.

	Theme	Subtheme
Theme 1.	Delay or loss to follow up resulting in severe sight loss in individuals with glaucoma or AMD	-
	*T1.1. “The original opticians referral was approximately six months ago according to the patient. Diagnosis of right extensive wet AMD with disciform scarring, beyond treatment criteria now, irreversible visual loss”* *T1.2. “Patient follow - up appointment has been delayed. Patient last seen November 2016 and follow - up appointment requested for 6 months”* *T1.3. “Lost to follow up Patient seen …. and 9 month follow up advised. Invalid disposal on patient [system]. Further appointment booked 2 years later when pressure control lost, and optic nerve condition deteriorated.”* *T1.4. “Her vision was down to Count fingers from 6 / 9 (Snellen chart) …, with progression of her visual field from glaucoma in both eyes, but resulting in loss of vision in her left eye.”* *T1.5. “Patient had a 6 / 12 [follow up].. in Ophthalmology Clinic booked …. [The appointment] was cancelled due to Annual Leave. The patient was not rebooked and instead put on [an appointment waiting list].….. This patient was not booked until they contacted Consultant directly …[5 months later].”*	
Theme 2.	Progression of glaucoma and AMD due to insufficient care	Inappropriate monitoring Inappropriate management
	*T2.1. “visual field test was not carried out at this visit, the most recent visual field test was carried out on XX November 2015”* *T2.2. “there is no cataract formation but a large choroidal mass clearly visible obscuring the posterior pole. The presumed diagnosis is a choroidal melanoma. The visual field defect corresponds to the extent of the lesion and is not a typical glaucomatous defect. The diagnosis was clearly not a cataract and should have been picked up much earlier. The visual field analysis in the left eye does not fit with a diagnosis of glaucoma and other reasons for the field loss should have been looked for.”* *T2.3. “…nurse requested visions and [optical coherence tomography] OCT prior [left eye intravitreal treatment] LE IVT, however only OCT was performed. Prepping outside IVT nurse dilated LE and did not realise vision and OCT was requested even though it was written on the clinic notes… was advised to continue with left IVT and review in 2 weeks time in virtual AMD appointment. Left OCT reviewed and large subretinal haemorrhage noted. Patient received Left IVT on [date] by the…. nurse. The patient was reviewed in the virtual AMD clinic ….[2 weeks later], left eye vision at Hand Movement (nil pinhole improvement)”* *T2.4. “…was not started on treatment at the time and booked for a 4-6mth review”* *T2.5. “Mr G stopped all previously prescribed glaucoma treatment and downgraded the diagnosis to ‘glaucoma suspect’ and requested follow up in 12 months.”*	
Theme 3.	System failures	Infrastructure and integration issues Accessing healthcare staff Referral incidents Protocol incidents Administrative and documentation incidents (Table S5) Lack of training/supervision (Table S5) IT issues (Table S5) Personnel issues (Table S5)
	*T3.1. “Subsequent appointments offered by the hospital in 2020 were cancelled by the hospital according to [the electronic records system].”* *T3.2. “…was not provided with the appropriate follow up appointment due to the lack of robust administration processes and training to ensure all staff appropriately actioned patients outcomes / follow up appointments on ..[the system] .”* *T3.3. “Patient contacted glaucoma department on multiple occasions regarding his continued postponement of appointments and worsening of vision however despite him expressing concern his appointments remained delayed”* *T3.4. “…was seen again a few times after the laser treatment and was found to have pressures of 36OD 41OS which is when she got listed for Left Phaco surgery as routine! No consultant saw the patient during this period! The patient needed urgent management for her Chronic Angle glaucoma which was deferred for no known reason”* *T3.5. “…spoken with Macular Unit and there is no trace of a Wet AMD referral having been received there”* *T3.6.“…had AMD changes since that date on OCT scan of left eye but was not referred to AMD clinic since.” T3.7. “Prior to 2015 this process was managed by the partial booking of follow - ups whereby patients would be on a worklist and safe, however this was stopped around that time and would be a contributing factor.”*	
Additional theme 4.	Adherence issues related to capacity or understanding and other patient factors (Table S5)	Adherence (Table S5) Impairment (Table S5) Factors outside patient’s control (Table S5) Confusion (Table S5) Other (Table S5)
Additional theme 5.	Impact of the COVID-19 pandemic (Table S5)	-

### Theme 1: delay or loss to follow up resulting in severe sight loss in individuals with glaucoma or AMD

Progression from delayed care featured in reports describing patients with glaucoma and AMD, in which severe vision loss occurred in 71.0% and 72.7% of incidents, respectively (*n* = 76 and *n* = 24). Of those with AMD, nearly half of the patients (*n* = 15, 45.4%) experienced loss of vision due to delayed care. For example, a patient with glaucoma was transferred to another clinic and a failure to detect visual field loss led to disease progression.

Delays in care, diagnosis or follow up resulted in a decrease in visual function or other worsening of their condition and were considered within this theme. The importance of disease progression due to patients being lost to follow up is highlighted by the impact on the patient, given that many eye conditions can result in irreversible loss of vision if left untreated. In one case where AMD was left untreated, loss of vision resulted (Table 4, quote T1.1).

In another example, a patient with glaucoma experienced a delayed follow up of years (rather than months) resulting in a dramatic vision loss, with severe consequences on the patient’s quality of life and their ability to maintain independence (Table 4, quote T1.2).

In many cases reported, visual acuity (VA) deteriorated to the most extreme levels of sight impairment at “count fingers”, “hand motion”, “light perception”, or “no light perception”, with permanent and complete loss of vision in extreme cases. Several incident reports described the progression of an eye condition when left untreated, and further conditions included diabetic retinopathy, and retinal detachment (Table 4, quote T1.3-4).

Several reports described patients being lost to follow up and causing a delay in accessing eye care for management, assessment or treatment. No further appointment was booked until they contacted the hospital and frequently patients suffered from this long wait (Table 4, quote T1.5).

### Theme 2: progression of glaucoma and AMD due to insufficient care

This theme describes incidents that report any form of insufficient care, such as staff not acting on progression of a disease or not assessing the patient adequately and includes both inappropriate monitoring as well as inappropriate management. Inappropriate monitoring occurred in more than half of the reports concerning patients with glaucoma (*n* = 27, 56.3%).

*Inappropriate monitoring* Key assessments (*n* = 8, 29.6%) and tests not being performed (*n* = 9, 33.3%) accounted for the majority of the cases involving insufficient monitoring resulting in glaucomatous progression. In 77.8% of cases, longitudinal monitoring was not appropriately undertaken and led to severe vision loss (Table 4, quote T2.1).

In one unusual report involving a misdiagnosis, which appeared to be an extreme combination of incidents. The patient presented with glaucoma and was prescribed topical treatment. On subsequent visits, it was thought that the glaucoma had progressed, and the patient was given additional eye drops to control intraocular pressure. The patient’s vision later deteriorated and attributed to the formation of cataract at first, and later a choroidal melanoma (Table 4, quote T2.2).

Other examples of insufficient care of patients with AMD included, for example, tests which were not performed or was interpreted incorrectly, resulting in progression of AMD, and inappropriate management was described in six cases (28.6%). In one example of a test not being performed for a patient with AMD, there was a delay in detecting loss of vision (Table 4, quote T2.3).

*Inappropriate management* Inappropriate management of patients with glaucoma and AMD was described in 21 and 12 cases, respectively, with a half and two-thirds, respectively, of these patients experiencing severe loss in vision. In some instances, no treatment was administered by staff, which occurred in 23.8% of glaucoma cases involving inappropriate management (Table 4, quote T2.4.). Treatment of glaucoma was stopped by staff in five reports, with two cases of severe vision loss (Table 4, quote T2.5).

### Theme 3: system failures

System failures included any incidents that resulted from staffing issues, protocol incidents or any infrastructure and integration issues, for example, staff capacity, a lack of clinical guidelines or problems with scheduling.

#### Infrastructure and integration issues

Glaucoma appointments being cancelled by the hospital (*n* = 10, 17.9%) and lack of capacity (*n* = 10, 17.9%) in hospitals were frequent causes (Table 4, quote T3.1.). Of the 10 cancellations made by hospitals, only three reports provided a reason about why the appointment was cancelled; one was due to staff on annual leave and two being COVID-19 related. Further, a patient with AMD was lost to follow up due to inadequate protocols, as well as a lack of training (Table 4, quote T3.2).

#### Accessing healthcare staff

Seven cases (7.7%) involved patients with glaucoma attempting to contact the hospital, with some informing the hospital of their symptoms, yet were still not seen in a timely manner. For example, a patient sought help for their deteriorating vision but was unable to secure an appointment (Table 4, quote T3.3).

#### Referral incidents

Reports describing referral incidents for patients with glaucoma occurred in 6.0% (*n* = 5) of cases, in which either no referral was made, or an incorrect referral time frame resulted in disease progression. In one example, a patient’s intraocular pressures became dangerously high due to a lack of proper referral at the appropriate time (Table 4, quote T3.4.):

For patients with AMD, reports describing referral incidents accounted for 20.5% (*n* = 8) of incidents describing disease progression due to system failures. All reports describing incidents in referrals resulted in patients experiencing severe vision loss (Table 4, quote T3.5.). Referrals not being made despite progression of the AMD also occurred (Table 4, quote T3.6).

#### Protocol incidents

Absent glaucoma management protocols were described frequently (*n* = 8, 42.1%), followed by inadequate protocols (*n* = 5, 26.3%), and changes in protocol (*n* = 4, 21.1%). Change in protocol referred to incidents in which changes to pre-existing protocols led to a patient safety incident (Table 4, quote T3.7).

## Discussion

The findings present a characterisation of eye-related incident reports occurring in a national population and include a qualitative analysis of the incidents resulting in severe harm and vision loss. Given the lack of research in patient safety in eye care, the results highlight the importance of and the need for an evidence base to underpin the learning from incidents that must be recognised in order to improve systems in eye care.

The key findings indicated that insufficient care was the most frequently reported type of incident, and such incidents caused the most severe harm to the patients. The most common contributory factor reported was continuity of care. Of the themes identified in those reports resulting in the most serious harm in which vision loss occurred, patients with glaucoma and AMD were impacted by delays or loss to follow up, insufficient care, and system failures. For example, unacceptably long delays in issuing follow up appointments, due to scheduling issues, resulted in significant and in some cases, potentially avoidable, disease progression. The findings highlight insufficient care, continuity of care, system failures and delayed diagnosis as priority areas for detailed evaluation and for the development of patient safety interventions, with a focus on patients with glaucoma and age-related macular degeneration.

The most frequently reported incident types included those from insufficient care and delayed diagnosis. For example, missed medications provided to patients with eye infections due to low staffing was categorised as insufficient care and delayed diagnosis were linked to lack of monitoring in some cases. Delays in eye care are evident in previous findings in patients experiencing sight loss, with lack of capacity cited as a major reason for delay [33]. Furthermore, delays and loss to follow up were identified in a study involving clinical record review in a hospital eye care setting, in which over half of incidents were a result of incomplete administrative processes [17]. Consistent with a review of safety incidents relating to ocular anti-vascular endothelial growth factor injections, delays were among the severe causes of harm identified, alongside intraocular inflammation or infection, with recommendations put forward involving system level planning, checklists and electronic records [34].

In the present study, a frequently reported contributory factor included issues with continuity of care, in which patients suffered vision loss due to appointment scheduling issues. Also, many patients suffered vision loss from appointments not being booked when requested, or appointments being cancelled and not rebooked. Consistent with previous findings, loss of medical records, failure to plan procedures, or transfer of care through paper referrals contributed to harmful outcomes [35]. Previous research has identified contributing factors to loss to follow up including patient non-attendance, hospital cancellation, and rescheduling and capacity issues [17]. Loss to follow up is a major problem in chronic diseases such as glaucoma when irreversible progression occurs before a patient becomes symptomatic, resulting in significant vision loss. Previous studies showed that 8% of glaucoma patients experience disease progression that could have been prevented with prompt follow-up [36]. A mixture of waiting list audits, triage guidelines, non-medical led clinics, a clear non-attendance policy, a specialist lead nurse role and a patient-focussed booking system was found to be effective in reducing loss to follow-up [37].

The findings from the reports included demonstrated that the severest level of harm most often affected patients with glaucoma and age-related macular degeneration. Given the high prevalence of these conditions, it is unsurprising that these findings mirror those in a similar population of patients [33]. In the present study, delays in management, assessment or treatment were a frequent outcome but mostly resulted in low or no harm. In other research, difficulties around delays due to appointment rescheduling in glaucoma services were associated with more serious harm [36] and the severity of unnecessary delays in urgent eye care settings has been highlighted [38].

System failures were evidenced in the data and included protocol errors, and staffing and infrastructure issues. Such challenges are mirrored by Davis et al. [17], but are not unique to eye care and have been demonstrated in other areas e.g. anaesthetic practice [39], endoscopic procedures [40] and medications safety [41]. Interventions addressing system failures, including implementation of protocol changes or standard operating procedures, were reported to reduce the number of incidents [12, 42, 43].

It is acknowledged that incidents are under-reported and may represent only a small proportion of the true problems in a system and can be limited in narrative content [30]. Like any incident reporting system, the NRLS and LFPSE are subject to potential reporting biases. The findings are essentially hypothesis-generating, and inductive, requiring testing and development in further studies and serve as potential areas of interest for improvement efforts in clinical practice.

## Conclusion

The findings highlight delays in patients receiving eye care as well as insufficient care, with severe levels of harm particularly impacting on patients with glaucoma and age-related macular degeneration. This study marks an important step in identifying priority issues for quality improvement and serves as a starting point for determining the areas of development. Given the restructuring of eye care services in the UK and increased clinical responsibility for some eye care clinicians, it is essential to promote a patient safety culture, such that everyone involved adheres to the safety guidelines toward improving the efficiency and effectiveness of patient pathways.

## Summary

### What was known before

- Despite patient safety being a global health priority for over two decades, there has been little focus on eye health safety.

### What this study adds

- The study represents the largest characterisation of eye-related incident reports from the National Reporting and Learning System and the NHS England Learn from Patient Safety Events system specifically focused on clinical assessment.
- Delays in patients receiving eye care as well as insufficient care, with severe levels of harm particularly impacting on patients with glaucoma and age-related macular degeneration are apparent potential priority issues for safety improvement.
- Care systems are complex and routine safety data can provide a critical steer to where and how they can be improved.

## Supplementary information

Supplementary Information

## Data Availability

The datasets generated during and/or analysed during the current study are not publicly available due to the nature of the data, and data sharing agreement with NHS England.
