# Supplementary material for: Patient safety in eye care: a multi-method analysis of reported incidents involving implementation of care and clinical assessment in England and Wales
Source: Eye (Lond). 2025 Feb 13;39(8):1486–94. doi: 10.1038/s41433-025-03669-6 (PMC12089528; doi:10.1038/s41433-025-03669-6)
Supplement: Supplementary file 1 — Supplementary Information [file 41433_2025_3669_MOESM1_ESM.docx]

**Supplementary Information**

Table S1. Search terms based on WHO ICD-11

'% conjunctiv%'

'% optician%'

'% optic%'

'% ocul%'

'% optomet%'

'% eye%'

'% glaucom%'

'% uveit%'

'% dacryocystit%'

'% periorbital cellulit%'

'% ophthal%'

'% orbital cellulit%'

'% ophthalm%'

'% ophthalmic%'

'% ophthalmology%'

'% ophthalmolog%'

'% retinal arterial occlusion %'

'% herpes zoster ophthalmicus %'

'% hypha%'

'% hyphema%'

'% hypopyon%'

'% papilloedem%'

'% papilledem%'

'% retina%'

'% retinal haemorrhag%'

'% retinal detach%'

'% vitreous detach%'

'% sclerit%'

'% temporal arterit%'

'% retinal break%'

'% retinal tear%'

'% cytomegalovir%'

'% candida retin%'

'% commotio retin%'

'% cornea%'

'% retinal vein occlu%'

'% keratoconjunctivitis%'

'% retrobulbar neurit%'

'% optic neurit%'

'% ocular rosac%'

'% rubeosis iri%'

'% squamous cell carcin%'

'% choroidal neovas%'

'% related macular degener%'

'% AMD %'

'% CNV %'

'% CRAO %'

'% CRVO %'

'% eyelid %'

'% hordeolum %'

'% chalazion %'

'% blephar%'

'% meibomian gland %'

'% lacrimal gland %'

'% orbit %'

'% cornea %'

'% keratitis %'

'% contact lens%'

'% iris %'

'% ciliary body %'

'% pupillary %'

'% staphyloma %'

'% choroid%'

'% retinopathy %'

'% high myopia %'

'% retinoschisis %'

'% vitreous %'

'% vitreoretinal %'

'% endophthalmitis %'

'% phthisis bulbi %'

'% neuroretinitis %'

'% third nerve palsy %'

'% 3^rd^ nerve palsy %'

'% fourth nerve palsy %'

'% 4^th^ nerve palsy %'

'% sixth nerve palsy %'

'% 6^th^ nerve palsy %'

'% oculomotor nerve palsy %'

'% keratopathy %'

'% cataract %'

Table S2. STROBE Statement checklist

|  | Item No. | Recommendation | Line  No. |
| --- | --- | --- | --- |
| **Title and abstract** | 1 | (*a*) Indicate the study’s design with a commonly used term in the title or the abstract | line 1-3 |
|  |  | (*b*) Provide in the abstract an informative and balanced summary of what was done and what was found | line 46-73 |
| Introduction | | | |
| Background/rationale | 2 | Explain the scientific background and rationale for the investigation being reported | line 92-116 |
| Objectives | 3 | State specific objectives, including any prespecified hypotheses | line 118-122 |
| Methods | | | |
| Study design | 4 | Present key elements of study design early in the paper | Line 127-133 |
| Setting | 5 | Describe the setting, locations, and relevant dates, including periods of recruitment, exposure, follow-up, and data collection | line 136-160 |
| Participants | 6 | (*a*) *Cohort study*—Give the eligibility criteria, and the sources and methods of selection of participants. Describe methods of follow-up  *Case-control study*—Give the eligibility criteria, and the sources and methods of case ascertainment and control selection. Give the rationale for the choice of cases and controls  *Cross-sectional study*—Give the eligibility criteria, and the sources and methods of selection of participants | line 136-160 |
|  |  | (*b*) *Cohort study*—For matched studies, give matching criteria and number of exposed and unexposed  *Case-control study*—For matched studies, give matching criteria and the number of controls per case | N/A |
| Variables | 7 | Clearly define all outcomes, exposures, predictors, potential confounders, and effect modifiers. Give diagnostic criteria, if applicable | line 136-160 |
| Data sources/ measurement | 8* | For each variable of interest, give sources of data and details of methods of assessment (measurement). Describe comparability of assessment methods if there is more than one group | line 136-206 |
| Bias | 9 | Describe any efforts to address potential sources of bias | line 172-175, line 401-406 |
| Study size | 10 | Explain how the study size was arrived at | Fig 1 |

Continued on next page

| Quantitative variables | 11 | Explain how quantitative variables were handled in the analyses. If applicable, describe which groupings were chosen and why | line 168-179 |  |
| --- | --- | --- | --- | --- |
| Statistical methods | 12 | (*a*) Describe all statistical methods, including those used to control for confounding | line 177-206 |  |
|  |  | (*b*) Describe any methods used to examine subgroups and interactions | line 185-194 |  |
|  |  | (*c*) Explain how missing data were addressed | line 173-175 |  |
|  |  | (*d*) *Cohort study*—If applicable, explain how loss to follow-up was addressed  *Case-control study*—If applicable, explain how matching of cases and controls was addressed  *Cross-sectional study*—If applicable, describe analytical methods taking account of sampling strategy | line 170-206 |  |
|  |  | (*e*) Describe any sensitivity analyses | line 185-206 |  |
| Results | | | |  |
| Participants | 13* | (a) Report numbers of individuals at each stage of study—eg numbers potentially eligible, examined for eligibility, confirmed eligible, included in the study, completing follow-up, and analysed | Fig 1 |  |
|  |  | (b) Give reasons for non-participation at each stage | Fig 1 |  |
|  |  | (c) Consider use of a flow diagram | Fig 1 |  |
| Descriptive data | 14* | (a) Give characteristics of study participants (eg demographic, clinical, social) and information on exposures and potential confounders | N/A |  |
|  |  | (b) Indicate number of participants with missing data for each variable of interest | N/A |  |
|  |  | (c) *Cohort study*—Summarise follow-up time (eg, average and total amount) | N/A |  |
| Outcome data | 15* | *Cohort study*—Report numbers of outcome events or summary measures over time | N/A |  |
|  |  | *Case-control study—*Report numbers in each exposure category, or summary measures of exposure | N/A |  |
|  |  | *Cross-sectional study—*Report numbers of outcome events or summary measures | Line 223-249 |  |
| Main results | 16 | (*a*) Give unadjusted estimates and, if applicable, confounder-adjusted estimates and their precision (eg, 95% confidence interval). Make clear which confounders were adjusted for and why they were included | N/A |  |
|  |  | (*b*) Report category boundaries when continuous variables were categorized | N/A |  |
|  |  | (*c*) If relevant, consider translating estimates of relative risk into absolute risk for a meaningful time period | N/A |  |

Continued on next page

| Other analyses | 17 | Report other analyses done—eg analyses of subgroups and interactions, and sensitivity analyses | | Line 251- 357 | |  |
| --- | --- | --- | --- | --- | --- | --- |
| Discussion | | | | | | |
| Key results | 18 | Summarise key results with reference to study objectives | line 365-372 | |  | |
| Limitations | 19 | Discuss limitations of the study, taking into account sources of potential bias or imprecision. Discuss both direction and magnitude of any potential bias | line 401-406 | |  | |
| Interpretation | 20 | Give a cautious overall interpretation of results considering objectives, limitations, multiplicity of analyses, results from similar studies, and other relevant evidence | line 373-400 | |  | |
| Generalisability | 21 | Discuss the generalisability (external validity) of the study results | line 373-400 | |  | |
| Other information | |  | | | | |
| Funding | 22 | Give the source of funding and the role of the funders for the present study and, if applicable, for the original study on which the present article is based | | line 426-427 | |  |

Table S3. Standards for Reporting Qualitative Research (SRQR) checklist

|  |  | |  |
| --- | --- | --- | --- |
|  | **Line no(s).** | |  |
| **Title and abstract** | |  | |
| **Title** - Concise description of the nature and topic of the study Identifying the study as qualitative or indicating the approach (e.g., ethnography, grounded theory) or data collection methods (e.g., interview, focus group) is recommended | line 1-3 | |  |
| **Abstract** - Summary of key elements of the study using the abstract format of the intended publication; typically includes background, purpose, methods, results, and conclusions | line 46-73 | |  |
|  |  | |  |
| **Introduction** | |  | |
| **Problem formulation** - Description and significance of the problem/phenomenon studied; review of relevant theory and empirical work; problem statement | Line 92-116 | |  |
| **Purpose or research questio**n - Purpose of the study and specific objectives or questions | line 118-122 | |  |
|  |  | |  |
| **Methods** | |  | |
| **Qualitative approach and research paradigm** - Qualitative approach (e.g., ethnography, grounded theory, case study, phenomenology, narrative research) and guiding theory if appropriate; identifying the research paradigm (e.g., postpositivist, constructivist/ interpretivist) is also recommended; rationale** | line 185-198 | |  |
| **Researcher characteristics and reflexivity** - Researchers’ characteristics that may influence the research, including personal attributes, qualifications/experience, relationship with participants, assumptions, and/or presuppositions; potential or actual interaction between researchers’ characteristics and the research questions, approach, methods, results, and/or transferability | line 196-197 | |  |
| **Context** - Setting/site and salient contextual factors; rationale** | line 141-166 | |  |
| **Sampling strategy** - How and why research participants, documents, or events were selected; criteria for deciding when no further sampling was necessary (e.g., sampling saturation); rationale** | line 141-166 | |  |
| **Ethical issues pertaining to human subjects** - Documentation of approval by an appropriate ethics review board and participant consent, or explanation for lack thereof; other confidentiality and data security issues | line 430-435 | |  |
| **Data collection methods** - Types of data collected; details of data collection procedures including (as appropriate) start and stop dates of data collection and analysis, iterative process, triangulation of sources/methods, and modification of procedures in response to evolving study findings; rationale** | line 141-206 | |  |
| **Data collection instruments and technologies** - Description of instruments (e.g., interview guides, questionnaires) and devices (e.g., audio recorders) used for data collection; if/how the instrument(s) changed over the course of the study | N/A | |  |
| **Units of study** - Number and relevant characteristics of participants, documents, or events included in the study; level of participation (could be reported in results) | Fig 1 | |  |
| **Data processing** - Methods for processing data prior to and during analysis, including transcription, data entry, data management and security, verification of data integrity, data coding, and anonymization/de-identification of excerpts | line 168-206 | |  |
| **Data analysis** - Process by which inferences, themes, etc., were identified and developed, including the researchers involved in data analysis; usually references a specific paradigm or approach; rationale** | line 185-206 | |  |
| **Techniques to enhance trustworthiness** - Techniques to enhance trustworthiness and credibility of data analysis (e.g., member checking, audit trail, triangulation); rationale** | line 196-206 | |  |
|  |  | |  |
| **Results/findings** | |  | |
| **Synthesis and interpretation** - Main findings (e.g., interpretations, inferences, and themes); might include development of a theory or model, or integration with prior research or theory | line 251-357 | |  |
| **Links to empirical data** - Evidence (e.g., quotes, field notes, text excerpts, photographs) to substantiate analytic findings | Table 4, Table S5 | |  |
|  |  | |  |
| **Discussion** | |  | |
| **Integration with prior work, implications, transferability, and contribution(s) to the field -** Short summary of main findings; explanation of how findings and conclusions connect to, support, elaborate on, or challenge conclusions of earlier scholarship; discussion of scope of application/generalizability; identification of unique contribution(s) to scholarship in a discipline or field | line 360-406 | |  |
| **Limitations** - Trustworthiness and limitations of findings | line 401-406 | |  |
|  |  | |  |
| **Other** | |  | |
| **Conflicts of interest** - Potential sources of influence or perceived influence on study conduct and conclusions; how these were managed | line 422-427 | |  |
| **Funding** - Sources of funding and other support; role of funders in data collection, interpretation, and reporting | line 426-427 | |  |
|  |  | |  |
|  |  | |  |
|  |  | |  |

Table S4. Data report from n=5000 incidents.

| **Incident Category Level 1** | **Count of incident** |
| --- | --- |
| **'Acute / general hospital'** | **4458** |
| 'Access, admission, transfer, discharge (including missing patient)' | 874 |
| 'Clinical assessment (including diagnosis, scans, tests, assessments)' | 520 |
| 'Consent, communication, confidentiality' | 226 |
| 'Disruptive, aggressive behaviour (includes patient-to-patient)' | 4 |
| 'Documentation (including electronic & paper records, identification and drug charts)' | 375 |
| 'Implementation of care and ongoing monitoring / review' | 609 |
| 'Infection Control Incident' | 324 |
| 'Infrastructure (including staffing, facilities, environment)' | 111 |
| 'Medical device / equipment' | 111 |
| 'Medication' | 212 |
| 'Other' | 170 |
| 'Patient abuse (by staff / third party)' | 7 |
| 'Patient accident' | 237 |
| 'Self-harming behaviour' | 4 |
| 'Treatment, procedure' | 674 |
| **'Ambulance service'** | **18** |
| 'Access, admission, transfer, discharge (including missing patient)' | 5 |
| 'Clinical assessment (including diagnosis, scans, tests, assessments)' | 3 |
| 'Consent, communication, confidentiality' | 1 |
| 'Implementation of care and ongoing monitoring / review' | 1 |
| 'Infrastructure (including staffing, facilities, environment)' | 1 |
| 'Medical device / equipment' | 1 |
| 'Other' | 2 |
| 'Patient accident' | 3 |
| 'Treatment, procedure' | 1 |
| **'Community and general dental service'** | **3** |
| 'Clinical assessment (including diagnosis, scans, tests, assessments)' | 1 |
| 'Implementation of care and ongoing monitoring / review' | 1 |
| 'Infection Control Incident' | 1 |
| **'Community nursing, medical and therapy service (incl. community hospital)'** | **352** |
| 'Access, admission, transfer, discharge (including missing patient)' | 25 |
| 'Clinical assessment (including diagnosis, scans, tests, assessments)' | 21 |
| 'Consent, communication, confidentiality' | 18 |
| 'Disruptive, aggressive behaviour (includes patient-to-patient)' | 3 |
| 'Documentation (including electronic & paper records, identification and drug charts)' | 19 |
| 'Implementation of care and ongoing monitoring / review' | 117 |
| 'Infection Control Incident' | 39 |
| 'Infrastructure (including staffing, facilities, environment)' | 3 |
| 'Medical device / equipment' | 1 |
| 'Medication' | 10 |
| 'Other' | 24 |
| 'Patient abuse (by staff / third party)' | 6 |
| 'Patient accident' | 28 |
| 'Self-harming behaviour' | 15 |
| 'Treatment, procedure' | 23 |
| **'Community optometry / optician service'** | **8** |
| 'Access, admission, transfer, discharge (including missing patient)' | 4 |
| 'Clinical assessment (including diagnosis, scans, tests, assessments)' | 1 |
| 'Documentation (including electronic & paper records, identification and drug charts)' | 2 |
| 'Medical device / equipment' | 1 |
| **'Community pharmacy'** | **13** |
| 'Medication' | 12 |
| 'Other' | 1 |
| **'General practice'** | **28** |
| 'Access, admission, transfer, discharge (including missing patient)' | 2 |
| 'Clinical assessment (including diagnosis, scans, tests, assessments)' | 6 |
| 'Consent, communication, confidentiality' | 1 |
| 'Documentation (including electronic & paper records, identification and drug charts)' | 3 |
| 'Implementation of care and ongoing monitoring / review' | 2 |
| 'Infection Control Incident' | 2 |
| 'Medication' | 10 |
| 'Treatment, procedure' | 2 |
| **'Learning disabilities service'** | **9** |
| 'Disruptive, aggressive behaviour (includes patient-to-patient)' | 2 |
| 'Implementation of care and ongoing monitoring / review' | 1 |
| 'Infection Control Incident' | 3 |
| 'Self-harming behaviour' | 2 |
| 'Treatment, procedure' | 1 |
| **'Mental health service'** | **111** |
| 'Access, admission, transfer, discharge (including missing patient)' | 6 |
| 'Clinical assessment (including diagnosis, scans, tests, assessments)' | 1 |
| 'Disruptive, aggressive behaviour (includes patient-to-patient)' | 10 |
| 'Implementation of care and ongoing monitoring / review' | 18 |
| 'Infection Control Incident' | 1 |
| 'Infrastructure (including staffing, facilities, environment)' | 6 |
| 'Medical device / equipment' | 1 |
| 'Medication' | 8 |
| 'Other' | 11 |
| 'Patient abuse (by staff / third party)' | 1 |
| 'Patient accident' | 21 |
| 'Self-harming behaviour' | 22 |
| 'Treatment, procedure' | 5 |
| **Grand Total** | **5000** |

Table S5. Additional subthemes in qualitative analysis.

| ***Theme 3: System failures*** |
| --- |
| **Administrative and documentation incidents** |
| Appointments not being booked occurred most frequently for both patients with glaucoma (n=6, 30.0%) and AMD (n=4, 25.0%). This included incidents in which clinicians requested for an appointment to be booked, which did not occur. For example: |
| *“…unclear why the appointment which was clearly requested but not subsequently booked in 2015 didn’t happen. The clinician filled in the outcome, however it appears that the administration team did not book the appointment or escalate that the appointment could not be booked and it was overlooked.”* |
|  |
| Five reports described documentation incidents in AMD, in which forms were completed incorrectly, or there was either inadequate documentation or none at all. An example of improper clinical documentation: |
| *“…don't consider this form to have been completed in full and no clear information added to the appointment / follow up area . Coding was entered for XXX and no other actions taken as form not clear what should happen next.”* |
|  |
| In many cases, follow up appointments were requested or discussed but not booked, with a failure to generate appointment letters or patients not receiving follow up appointments despite chasing. Many patients were due clinical review, but were not placed on a booking renewal or were left on a waiting list. In some cases, patients cancelled appointments, which were then not rebooked, with no further appointments offered. |
| “*Patient seen ….. with glaucoma and retinal vein occlusion …. [Two month] review requested. Patient received no follow up despite chasing.”* |
|  |
| **Lack of training/supervision** |
| Insufficient training incidents described reports where staff were not trained in other specialities within eye care and hence, unable to identify other abnormalities. One incident described both a lack of training and supervision occurring at the same time: |
| *“The incident has highlighted a probable competency issue in relation to diagnosing an unexpected serious comorbidity at the posterior pole. This raises issues in terms of the level of supervision provided to non - medical staff in the glaucoma clinic, and for the non - medical staff themselves , knowing when to seek advice.”* |
|  |
| **IT issues** |
| Problems with technology formed a minority of system failures leading to glaucomatous progression (n=3, 3.3% of all system failures). In one incident, a staff member was unable to access the results of a visual field test between different hospital sites and therefore unable to identify deterioration in visual function: |
| *“[Staff Name] was unable to access visual fields taken in 2013 from XXX , because of lack of connectivity between XXX and XXX systems for Forum software and therefore on [present date] when at XXX, was able to compare visual fields taken in 2013 with visual fields taken [present date]”* |
|  |
| **Personnel issues** |
| Severe vision loss occurred in four glaucoma cases as a result of staff being on annual leave (n=1, 25.0%) or the hospital being understaffed (n=3, 75.0%). The following is an example a patient’s glaucoma appointment being cancelled due to staff being on leave: |
| *“a 9 / 12 follow up appointment was requested . The patient was given an appointment … but this was cancelled by hospital due to annual leave”* |
|  |
| ***Theme 4: Adherence issues related to capacity or understanding and other patient factors*** |
| Progression of glaucoma and AMD as a result of patient factors occurred due to various reasons including patients not adhering to their treatment, in some cases, related to the patient’s capacity or understanding or their refusal of procedures. Adherence issues were described for 24 patients with glaucoma. Factors outside the patient’s control was described in 6 reports of patients with AMD. |
|  |
| **Adherence** |
| Poor adherence with glaucoma medication accounted for 9 cases, with eight of these resulting in severe vision loss. There was an incident in which a patient was continually missing appointments and did not adhere to the use of the prescribed glaucoma eye drops despite constant reminders from healthcare staff. This patient later suffered from severe vision loss: |
| *“…level of vision was noted in February 2021 when he was also noted to not be using any of his glaucoma medications . Glaucoma medications were re - issued by the … doctor … [in Jan 2020], … , and it was noted that he had not been using his prescribed glaucoma medications . He was similarly re - issued these and urgently referred to the glaucoma clinic , which he failed to attend on two successive occasions [in 2020] . . The last glaucoma clinic appointment that he did attend was …[in 2019]… It was noted at that visit that he was also not using the prescribed glaucoma medications and was re - issued them and explained the risk of losing vision if left uncontrolled . A two - month review with the glaucoma consultant was requested following this visit in August 2019, but the patient was next seen …[in 2020] when he self - presented with loss of vision…”* |
|  |
| Two cases reported incidents of patients ceasing glaucoma medication. Both incidents involved patients who were taking eye drops for glaucoma, before ceasing to use them due to side effects or loss of vision. For example: |
| *“When his right vision went off completely, he stopped his topical anti-glaucoma medications.”* |
|  |
| **Impairment** |
| Alzheimer’s and dementia was described in three glaucoma cases. This resulted in the patient being non-adherent with examinations or medication. In such reports, it was not acknowledged whether special assessment techniques were implemented to help patients with communication difficulties. For example: |
| *“Patient has Alzheimer and glaucoma and difficult to assess due to limited compliance with necessary tests”* |
|  |
|  |
| **Factors outside patient’s control** |
| Uncontrolled glaucoma despite a patient being on treatment was described in 4 cases. An example of a patient with glaucoma experiencing loss of vision: |
| *“…then consultant put him into waiting list for rt eye trabeculectomy . but he rang … to let the pre op team know that he lost the vision in right eye and did not want the surgery . .”* |
|  |
| In another case, staff were unable to obtain retinal images in a patient with AMD, which were essential for clinical monitoring and management decisions: |
| *“Medical Photographer staff member were unable to capture macular fundus photograph and OCT to the Left eye due to poor fundus view.”* |
|  |
| **Confusion** |
| Older individuals may be required to attend numerous health appointments and have varying levels of support to attend their appointments. One incident of confusion described a patient with both AMD and glaucoma. The patient was previously being treated at another hospital, before transferring to a new area, where she was then supposed to undergo AMD treatment. However, the patient mistakenly attended the wrong clinic: |
| *“Patient does not remember being told she will have treatment and does not know what has happened . She was confusing the glaucoma and AMD visits . .”* |
|  |
| **Other** |
| A patient who delayed their glaucoma surgery was described in one report. The clinician and patient both agreed on a fixed date for follow up. However, this appointment was delayed, despite the clinician’s instructions. This eventually led to significant disease progression resulting in severe vision loss in the affected eye: |
| *“…left trabeculectomy was offered and recommended. She decided to defer this due to work commitments and I advised that there was a risk in delaying surgery, which she understood. We agreed an appointment in 2 months (August 2022), … rated as red and """"not to wait"""" …. She contacted XXX in September 2022 as she had been due in August. She was told that she was on a waiting list. She felt visual changes in December and ache around left eye. She did not access XXX or her own optometrist. She contacted XXX again in December and an appointment made for … Feb 2022. At this review she had a drop in her left visual acuity to 3/60, IOPs of 46, dense pigmentation of the anterior chamber angle, a left RAPD and significant disc cupping of 0.95 cup to disc ratio. There was a reduction in her field mean deviation from -19.46 to -31.19”* |
|  |
| There was one incident of an unusual presentation in AMD. A patient was undergoing treatment and developed retinal scarring, which the consultant claimed was related to the side effects of the treatment: |
| *“…too late as scar tissue had formed and there was permanent visual loss . He also said to me ( not to the patient though ) that this may have been a side effect from the lucentis . . I rang our …[pharmaceutical company] rep to ask her opinion as I had not heard of this before . She was on holiday and asked her colleague to look into it for her . Her colleague automatically rang the Medical Informations department at …[pharmaceutical company] and said that I would be contacting them .”* |
|  |
| ***Theme 5: Impact of the COVID-19 pandemic*** |
| A total of 18 incidents describing COVID-19 were reported and included incidents in communication, delays and scheduling issues. Of the 18 reports, 11 described patients with glaucoma, with the remaining describing patients with AMD, diabetic retinopathy, cataract, uveitis and optic nerve abnormalities. Telephone consultation was described in three incidents, with one incident resulting in miscommunication between the patient and staff. This later led to severe vision loss, as the staff were not able to identify the need for an urgent follow up: |
| *“ ….appointment cancelled due to COVID-19 which could have identified the need for a glaucoma follow - up appointment ... Call from patient on 7th August 2020 did not raise any concerns that an urgent follow - up appointment for glaucoma had been missed only that of the need for ..[another]… appointment that had previously been cancelled due to COVID-19.”* |
|  |
| Another incident involving telephone consultation described a patient who was due to be seen at an in-person clinic, but due to COVID-19, the patient had a telephone consultation instead. Despite the clinician restarting treatment, the patient still experienced severe vision loss: |
| *“Glaucoma treatment stopped by GP some time during lost period . ( no idea why ) Referred back at start of the year . Seen in glaucoma technician clinic and was due to be seen in glaucoma main clinic in April so had telephone consultation then at that time due to COVID Crisis . Recommenced remotely on antiglaucoma medication”* |
